# Impacts of Climate Change and Heat Stress on Farmworkers' Health: A Scoping Review

**DOI:** 10.3389/fpubh.2022.782811

**Published:** 2022-02-08

**Authors:** Moussa El Khayat, Dana A. Halwani, Layal Hneiny, Ibrahim Alameddine, Mustapha A. Haidar, Rima R. Habib

**Affiliations:** ^1^Department of Environmental Health, Faculty of Health Sciences, American University of Beirut, Beirut, Lebanon; ^2^Saab Medical Library, American University of Beirut, Beirut, Lebanon; ^3^Department of Civil and Environmental Engineering, Maroun Semaan Faculty of Engineering and Architecture, American University of Beirut, Beirut, Lebanon; ^4^Department of Agriculture, Faculty of Agricultural and Food Sciences, American University of Beirut, Beirut, Lebanon

**Keywords:** agricultural workers, farmworkers, climate change, global warming, heat stress, heat exposure, occupational health

## Abstract

Due to the continuous rise of global temperatures and heatwaves worldwide as a result of climate change, concerns for the health and safety of working populations have increased. Workers in the food production chain, particularly farmworkers, are especially vulnerable to heat stress due to the strenuous nature of their work, which is performed primarily outdoors under poor working conditions. At the cross-section of climate change and farmworkers' health, a scoping review was undertaken to summarize the existing knowledge regarding the health impacts associated with climate change and heat stress, guide future research toward better understanding current and future climate change risks, and inform policies to protect the health and safety of agricultural workers. A systematic search of 5 electronic databases and gray literature websites was conducted to identify relevant literature published up until December 2021. A total of 9045 records were retrieved from the searches, of which 92 articles were included in the final review. The majority of the reviewed articles focused on heat-related illnesses (*n* = 57) and kidney diseases (*n* = 28). The risk factors identified in the reviewed studies included gender, dehydration, heat strain, wearing inappropriate clothing, workload, piece-rate payment, job decision latitude, and hot environmental conditions. On the other hand, various protective and preventive factors were identified including drinking water, changing work hours and schedule of activities, wearing appropriate clothing, reducing soda consumption, taking breaks in shaded or air-conditioned areas, and increasing electrolyte consumption in addition to improving access to medical care. This review also identified various factors that are unique to vulnerable agricultural populations, including migrant and child farmworkers. Our findings call for an urgent need to expand future research on vulnerable agricultural communities including migrant workers so as to develop effective policies and interventions that can protect these communities from the effects of heat stress.

## Introduction

Climate change has led to a significant increase in global average temperatures; temperatures on average have increased by about 1.1°C since pre-industrial times (1). In addition, the intensity, frequency, and duration of heatwaves have been rapidly increasing around the globe (2). Nineteen out of the 20 warmest years on record have occurred since 2000 (3). Between 1950 and 2017, a majority of the world's regions has been experiencing at least one extra day of heatwave per decade (4). It is predicted that the trend of rising temperatures and the occurrence of more frequent, longer, and more intense extreme heat events will continue in the future (5, 6). By the end of the century, 1.2 billion people are expected to be affected by heat stress, which is four times the number of people that are currently affected by extreme heat (7). Exposure to high temperatures can result in various adverse health impacts. These range from acute health effects to more severe and chronic conditions and in extreme circumstances these impacts can even lead to death (8–10). The consequences of global warming are expected to have disproportionate impacts on countries and communities that have contributed the least to the problem and are the least able to adapt to heat stress (11).

Compared to the general public, working populations, including farmworkers, are more susceptible to heat stress. The combined effect of the metabolic heat produced internally from heavy physical activity and the external heat from the surrounding environment contributes to the high risk of heat stress among workers (12, 13). Workers engaged in strenuous work at temperatures >35°C are likely to experience heat stress (14). In addition, some occupations require workers to wear protective clothing, which can inhibit sweat evaporation and normal heat dissipation thus exacerbating their risk of heat stress (15). Furthermore, workers who perform their tasks outdoors are vulnerable to heat stress due to prolonged exposure to solar radiation, while workers in indoor settings can also be exposed to heat exposure from the heat generated from work processes or equipment (16). Recent publications warn that climate change is expected to intensify the duration and magnitude of occupational heat stress (17). Projections for the year 2030 estimate the loss of 880,000 work-life-years due to occupational heat stroke mortality in both indoor and outdoor workplaces (18). The impact of rising heat in the workplace is likely to affect the implementation of the 2030 Sustainable Development Goals (SDGs), including those related to poverty, food security, health, decent work, and economic growth and inequality (19).

The agricultural workforce has been recognized as a vulnerable occupational group with an increased risk of adverse health outcomes from rising global temperatures (20, 21). In fact, occupational heat-related mortality is 35 times higher among agricultural workers compared to workers from other industries (22). Agricultural workers are often exposed to hot environmental conditions, which exceed international standards (23–25). In addition, the literature identified several work-related factors that exacerbate the risk of heat stress among agriculture workers, which include (1) performance of intensive manual labor (26), (2) piece-rate payment that encourages agricultural laborers to work beyond their physical limits and avoid taking breaks to rest or hydrate (27, 28), and (3) the lack of control over workplace health and safety practices and adequate access to water, shade, or rest breaks (29, 30). In addition to the harsh working conditions, agricultural workers also face poor living conditions including a lack of access to clean drinking water, adequate housing, and basic social and health care services (31, 32). Furthermore, the majority of farmworkers live in low socioeconomic conditions, whereby a study found that the levels of extreme poverty are four times higher among agricultural workers as compared to non-agricultural workers (33).

Globally, migrants account for an increasing share of the agricultural workforce, with 16.7 million migrants engaged in the agricultural sector worldwide (34). The migration of agricultural workers is driven by various social, economic, and environmental factors that are likely to get exacerbated with climate change, leading to a greater flow of migrants especially from low-income countries (35). Migrant agricultural workers often face unique vulnerabilities that affect their health and wellbeing and increase their risks of heat-related health outcomes. Migrant agricultural workers are subjected to unsafe working conditions and increased workplace harassment (36–38). In addition, these workers are not protected by labor regulations and are not represented in labor unions; they often also face language and cultural barriers and lack access to social security (39).

Several interlinkages between climate change, farmworkers' health, and food production have been identified in the literature. Climate change is expected to impact agricultural production, leading to higher levels of poverty and food insecurity among agricultural workers (40). Furthermore, it is projected that farmworkers will only be able to work for a fraction of the hours they currently work due to rising heat stress. This can lead to a further decline in agricultural production (41). One estimate projects that the agricultural sector will account for 60% of the global working hours lost due to heat stress by the year 2030 (19). Moreover, heat related health outcomes among agricultural workers will also reduce their productivity (42). Although agricultural workers play a critical role in food production, studies have shown that they are highly food insecure; they endure precarious working conditions and earn low wages that hinder access to food (43). In addition, the loss of productivity is expected to exacerbate their food insecurity, especially in small-scale and subsistence agriculture (44).

Heat-related Illnesses (HRI) are the most common health effects experienced by agricultural workers as a result of their prolonged exposure to high temperatures (30, 40–42). HRI are a continuum of diseases, ranging from mild symptoms such as heavy sweating, dizziness, fatigue, vomiting, headaches, and muscle cramps to more severe conditions such as life-threatening heat strokes (12). Occupational heat exposure and dehydration have also been linked to an epidemic of chronic kidney diseases referred to as “chronic disease of unknown etiology (CKDu)” among agricultural communities in several hot regions including Central America and Southeast Asia (43–45). In addition, agricultural workers laboring in hot conditions are at an increased risk for workplace injuries due to increased fatigue, reduced alertness, deterioration in psychomotor abilities, and loss of concentration (46, 47).

With the continuous increase in global temperatures and the severity of its impact on the health and safety of the agricultural workforce globally, a scoping review is needed to summarize the existing knowledge regarding the impact of climate change, particularly extreme heat exposure on farmworkers' health and the available prevention strategies to alleviate these impacts on farmworkers. Thus, the aims of this review are to: (1) summarize the available evidence on the effects of climate change on farmworkers' health with a focus on heat-related illnesses, (2) identify the risk factors for heat-related illnesses among farmworkers, and (3) review the preventive measures that are used to minimize heat stress exposure among farmworkers. To the best of our knowledge, no other reviews has been carried out to study the effects of climate change and heat stress on the health and safety of farmworkers. This work also informs policymakers to develop more effective policies and programs to protect vulnerable farming communities from the impacts of climate change.

## Methods

### Search Strategy

To meet our objectives and map the available literature on this topic, we conducted a scoping review following the PRISMA extension for Scoping Reviews (PRISMA-ScR) (48). In line with this protocol, we developed a search strategy using a combination of controlled vocabulary such as Medical Subject Headings (MeSH) and keywords for each of the 5 selected electronic databases: Medline, Embase, Scopus, CINAHL, and Web of Science. The search strategies are detailed in the Supplementary Material (Supplementary Table S1). Two concepts were used in our search strategy: (1) heat exposure and (2) agricultural setting.

- Keywords used for heat exposure included: extreme temperature, thermal comfort, thermal sensation, global warming, heatwave, and climate variability.
- Keywords used for agricultural setting included: farmworker, farmer, harvester, grower, agriculturalist, and agricultural worker.

No restrictions were applied on the year of publication and language to capture the maximum number of articles that have addressed the topic in the literature. Specific health outcomes were also not included in the search strategy to capture all heat-related health effects covered in the medical and public health literature that was published in the selected databases.

In addition, the websites of international agencies (ILO, WHO, and UNDP) and agricultural databases (AGRIS FAO and AgEcon) were manually searched to identify relevant articles. The reference lists were also checked in the gray literature to find additional studies that could be relevant for the review. The database search was conducted on December 6, 2021 and the gray literature search was conducted on December 24, 2021.

### Eligibility Criteria

We aimed to reduce the restrictions in the eligibility criteria so as to explore most of the available literature on heat-related health outcomes among farmworkers. We considered as eligible for this scoping review the following:

- Outcome of interest: Studies assessing: (1) Health outcomes related to heat exposure; (2) Risk and protective factors associated with health outcomes; and (3) Preventive measures. We excluded studies assessing outcomes not related to heat stress and studies that calculate the effects with simulations or models instead of actual measurements in humans.
- Population of interest: Farmworkers of all age groups.
- Context of interest: Studies assessing the impact of occupational heat exposure on workers' health and safety.
- Study design: Primary studies including quantitative and qualitative methods. We excluded editorials, commentaries, letters to the editors, reviews, reports, and conference abstracts.

### Selection Process

Two stages for the screening and selection of articles were carried out independently and in duplicates by two reviewers (M.K. and D.A.H.) and guided by the pre-established eligibility criteria. Calibration exercises were conducted by the 2 reviewers (M.K. and D.A.H.) to assess and refine, if necessary, the screening questions and ensure the validity of the selection process. The records that were deemed eligible for inclusion by the two reviewers during the title and abstract screening phase were screened at the full-text stage. Disagreements about inclusion in the full-text screening were resolved through discussion until consensus was reached between both reviewers. If consensus could not be reached, a third reviewer (R.R.H.) was consulted to resolve the disagreement.

### Data Abstraction and Analysis

Data were extracted from the included articles by one of the reviewers (M.K.), using a pre-established data abstraction form. This form was developed and reviewed by the authors of this review. A calibration exercise was conducted by the 2 reviewers (M.K. and D.A.H.) before the data abstraction phase. The calibration allowed the 2 reviewers to assess and refine the fields/data items in the form and to ensure the validity of the data abstraction process (i.e., the same information is extracted by both reviewers). Information relating to the study characteristics and the findings were extracted from each selected study. These included: (1) first author and year of publication, (2) study location and region, (3) study design, (4) study population and sample size, (5) heat exposure metric, (6) period of heat exposure and study duration, (7) methods of data collection (8) data analysis, (9) heat-related health outcome, (10) risk factors, (11) protective factors and (12) preventative measures.

Data on health outcomes, risk and protective factors, and preventative measures were descriptively summarized and analyzed to identify research gaps. Furthermore, statistically significant risk and protective factors from quantitative studies and all factors from qualitative studies were classified into superordinate categories.

## Results

**Figure 1:**
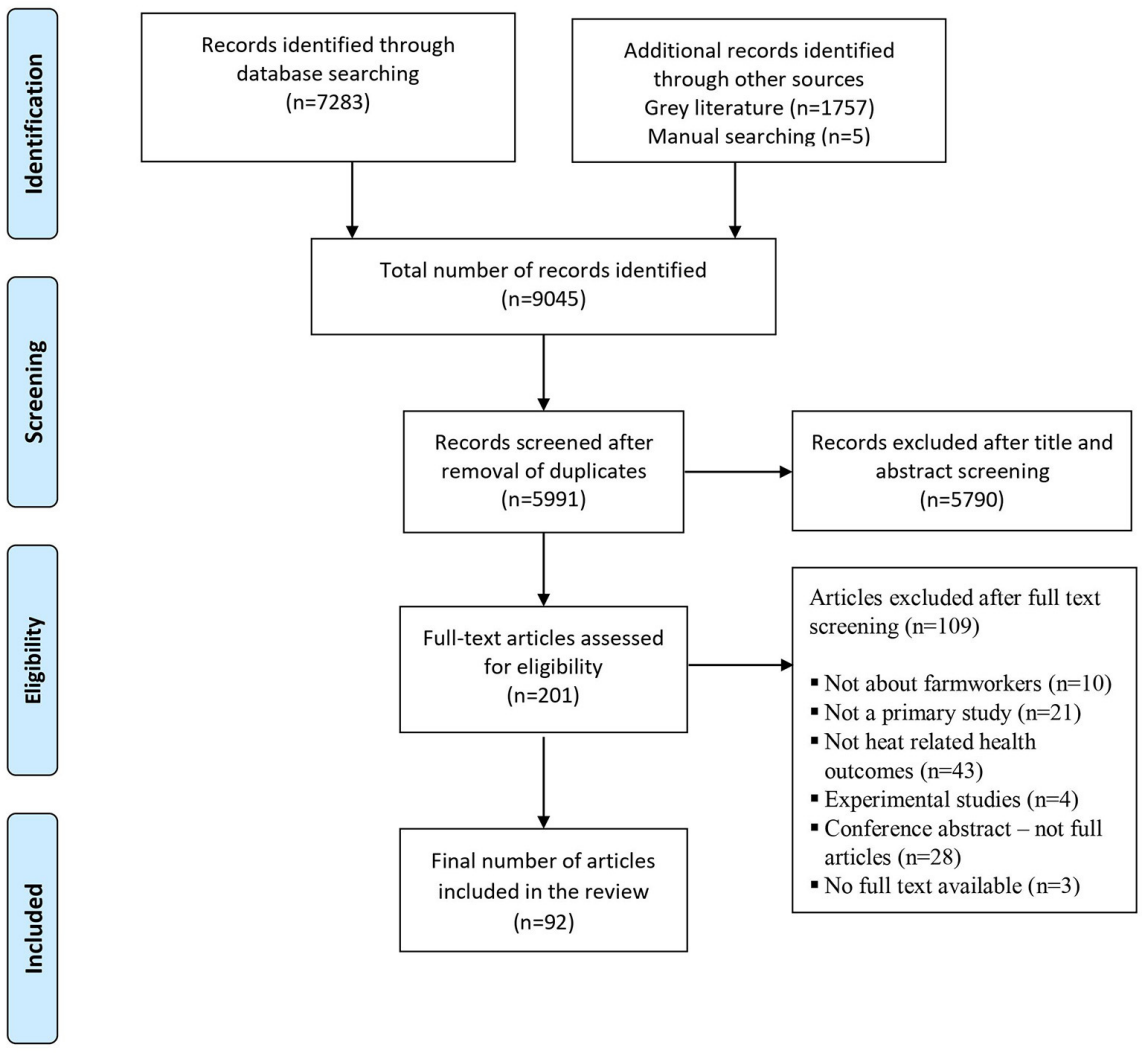
Flow chart of selection process for included studies.

The selection process of the articles is presented in Figure 1 using the PRISMA flow diagram. 7,283 records were identified from the database search, while an additional 1,757 records were identified through searching of gray literature. In addition, 5 records were identified through manual searching. 5,991 records remained for the title and abstract screening stage after duplicate removal. After completing the title and abstract screening of the identified records, 201 articles remained for full-text screening. One hundred nine records were excluded for various reasons as listed in the flow diagram and 92 records were included for the final review.

### Descriptive Characteristics of Included Studies

**Figure 2:**
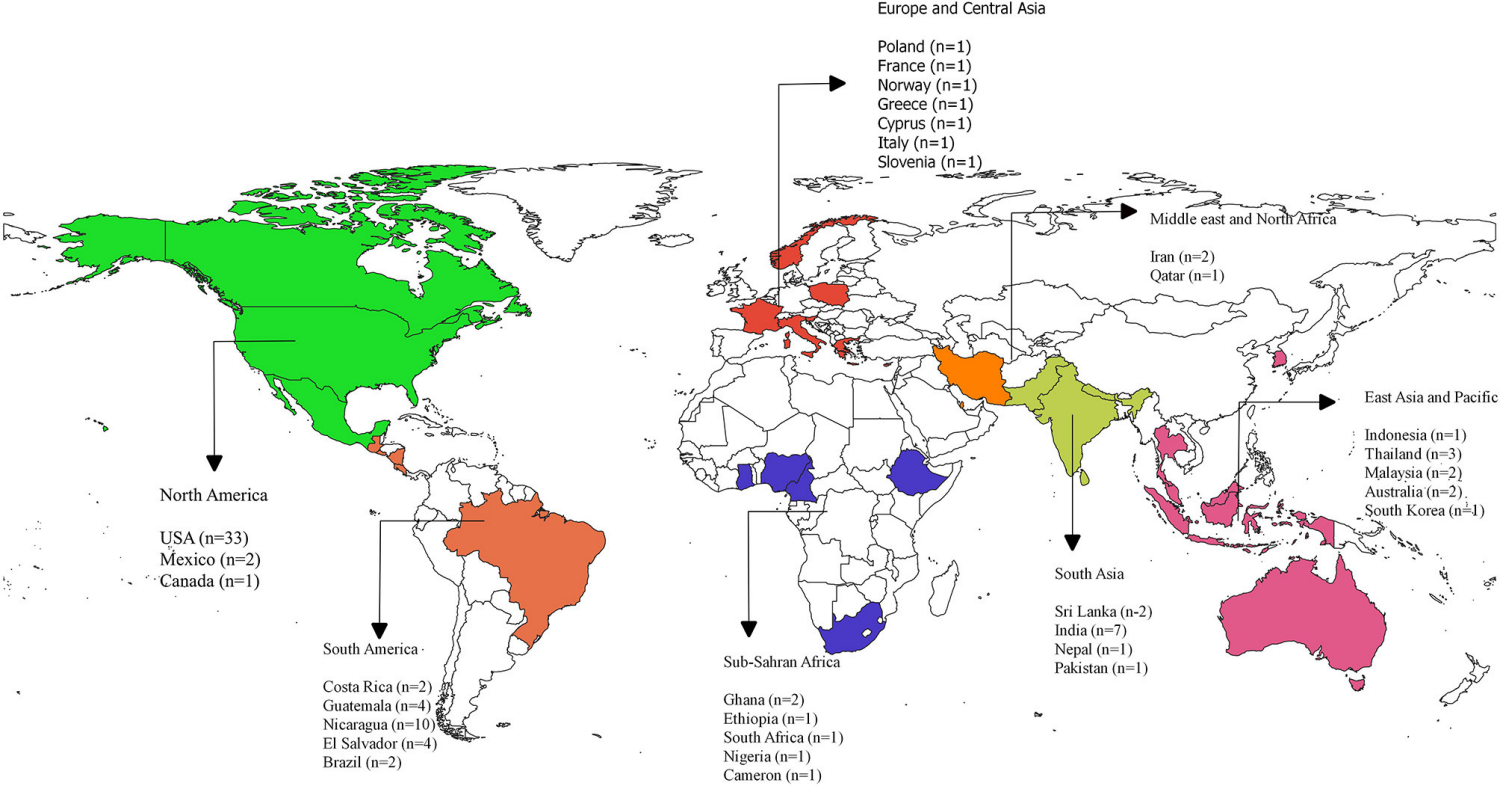
Geographical distribution of the included studies. “*n*” denotes the number of publications in each country; USA, United States of America.

Table 1 presents the descriptive characteristics of the studies included in this review. The results reveal an increase in the number of articles published on the topic over the last couple of years. The highest number of publications was in 2019, with 16 articles published that year. The majority of reviewed articles were quantitative studies, which included cross-sectional (*n* = 65; 71%), longitudinal (*n* = 15; 16%) and comparative studies (*n* = 1; 1%). The remaining articles used qualitative (*n* = 9; 10%) and mixed methods designs (*n* = 2; 2%). In addition, the majority of the reviewed articles used questionnaires (*n* = 69; 75%), followed by physiological measurements (*n* = 38; 41%) and biological sampling (*n* = 36; 39%) as data collection tools. While some studies used interview/focus groups (*n* = 7; 8%), the rest of the articles used observations (*n* = 3; 3%). The majority of the included articles focused on agricultural workers/farmworkers (*n* = 54; 59%) and sugarcane farmers/cutters (*n* = 23; 25%). While the remaining articles studied pesticide applicators (*n* = 1;1%), nursery workers (*n* = 1; 1%), sheep shearers (*n* = 1; 1%), tractor driver (*n* = 1; 1%), forestry (*n* = 2; 2%), harvesters (*n* = 4; 4%), fernery workers (*n* = 3; 3%), crop workers (*n* = 1;1%), horticulture workers (*n* = 1;1%) and vineyard workers (*n* = 1; 1%). The majority of the reviewed studies were conducted in North America (*n* = 36; 39%), followed by South America (*n* = 22; 24%), and South Asia (*n* = 11; 12%). The remaining articles were distributed among East Asia and the Pacific (*n* = 9; 10%), Europe and Central Asia (*n* = 6; 7%), the Middle East and North Africa (*n* = 3; 3%), and Sub-Saharan Africa (*n* = 6; 7%). Figure 2 presents a map of the geographical distribution of the studies by country.

**Table 1:** Descriptive characteristics of the included articles.

**Characteristics**	***N***	**%**
**Study design**
*Quantitative studies*
Cross-sectional	65	71
Longitudinal	15	16
Comparative	1	1
Qualitative	9	10
Mixed methods	2	2
**Data collection methods**
Questionnaire	69	75
Biological sampling	36	39
Physiological measurements	38	41
Interview/focus group	7	8
Observation	3	3
**Study population**
Forestry workers	2	2
Farmworkers/agricultural workers	54	59
Fernery workers	3	3
Tractor driver	1	1
Nursery workers	1	1
Horticulture	1	1
Harvesters	4	4
Crop workers	1	1
Pesticide applicators	1	1
Sheep shearers	1	1
Sugarcane workers/cutters	23	25
Vineyard workers	1	1
**Region**
North America	36	39
South America	22	24
Middle East and North Africa	3	3
South Asia	11	12
East Asia and Pacific	9	10
Europe and Central Asia	6	7
Sub-Saharan Africa	6	7

The types of heat-related health outcomes assessed in the included studies are presented in Table 2. The most common heat-related health outcome assessed was HRI (*n* = 57; 62%). Twenty-seven out of 57 of these studies relied on self-reported HRI symptoms (25, 27, 30, 40–42, 49–69), while 21 measured various physiological parameters such as core body temperature, skin temperature, heart rate, blood pressure, changes in body weight, or urine specific gravity (61, 68, 70–88). Another commonly assessed heat-related health outcome was kidney disease (*n* = 28; 30%). Several of these studies determined the prevalence of Chronic Kidney Disease (CKD), (89–91) Acute Kidney Injury (AKI) (28, 92–97), and Incident Kidney Injury (IKI) among agricultural workers (98–100). The remaining articles assessed other health outcomes including cardiorespiratory symptoms (*n* = 1; 1%), respiratory diseases (*n* = 2; 2%), injuries (*n* = 1; 1%), reproductive health (*n* = 1; 1%), skin disorders (*n* = 1; 1%), and diabetes and hypertension (*n* = 1; 1%).

**Table 2:** Heat-related health outcomes of included studies.

**Heat-related outcomes**	***N***	**%**
Heat-related illnesses	57	62
Kidney diseases	28	30
Cardiorespiratory symptoms	1	1
Respiratory diseases	2	2
Injuries	1	1
Skin disorders	1	1
Reproductive health	1	1
Diabetes and hypertension	1	1

A variety of heat exposure metrics were used to measure heat stress in the included studies. Overall, 41 articles employed some form of heat exposure metric. Some of these studies used simple ambient weather metrics, including temperature (*n* = 8; 9%) and relative humidity (*n* = 4; 4%). In addition, composite indices accounting for temperature and other weather parameters were used such as the Wet Bulb Globe Temperature (WBGT) index (*n* = 33; 36%), heat index (*n* = 6; 7%), heat stress index (*n* = 2; 2%), humidex (*n* = 2; 2%) and universal thermal climate Index (*n* = 2; 2%).

### Factors Associated With Heat-Related Outcomes

Seventeen articles reported risk or protective factors for HRI, 15 of which reported statistical measures of association, while 3 reported potential factors without a statistical analysis to determine the significance of the association with HRI. In addition, 23 out of 28 studies assessing kidney disease identified various risk or protective factors.

#### Factors Associated With HRI

##### Work Organization and Management

###### Workload.

Three studies assessed the association between core body temperature and workload that was measured using an accelerometer among U.S. farmworkers (Table 2) (74, 75, 79). Two of these studies found that there was a 12% increase in the odds of reaching or exceeding 38°C for every 100 kilocalories of energy expended among fernery workers (74, 79). Similarly, Vega-Arroyo et al. (75) found that an increase in the work rate was associated with higher core body temperatures (β = 0.006, 95% CI: 0.004, 0.009).

###### Work Duration.

One study found that work duration was associated with greater cardiac strain among vineyard workers (β: 0.219; 95% CI: 0.033–0.405) (85).

###### Payment Method.

Three studies identified piece-rate payment as a potential risk factor for HRI (Table 3) (27, 101, 102). However, only one study measured the association between HRI and piece-rate payment and found a significant association (OR: 6.20; 95% CI: 1.11–34.54) (27). In contrast, one study did not find a significant association between piece-rate work and HRI (60).

**Table 3:** Risk and protective factors associated with HRI.

**Factors**	**Risk factor**	**Protective factor**	**No significant association**	Comment^*^
**Work organization and management**				
Workload	Mac (79) Mac et al. (74) Vega-Arroyo et al. (75)			Suggested evidence of increased risk of HRI
Payment method	Crowe et al. (102) Spector et al. (27) Luque et al. (101)		Arcury et al. (60)	Suggested evidence of increased risk of HRI
Job decision latitude	Hansen et al. (29) Lam et al. (103)			Suggested evidence of increased risk of HRI
Work safety climate	Wilmsen et al. (104)			Limited evidence of increased risk of HRI
Task duration	Grimbuhler and Viel (85)			Limited evidence of increased risk of HRI
**Behavioral factors**				
Rest in shaded area	Riccò et al. (63)	Fleischer et al. (30)		Limited evidence of reduced risk of HRI
Extra breaks		Fleischer et al. (30) Arnold et al. (51)	Spector et al. (27) Riccò et al. (63)	Inconsistent or conflicting evidence
Change work hours and activities		Mirabelli et al. (40)		Limited evidence of reduced risk of HRI
Reduce soda consumption		Fleischer et al. (30)		Limited evidence of reduced risk of HRI
Fluid intake			Kiatkitroj et al. (69)	Limited evidence of reduced risk of HRI
Wear excessive clothing		Kiatkitroj et al. (69)		Limited evidence of reduced risk of HRI
**Accessibility to heat prevention and treatment**				
Culture and language	Hansen et al. (29) Lam et al. (103)			Suggested evidence of increased risk of HRI
Access to toilet	Spector et al. (27)			Limited evidence of increased risk of EHI
Access to medical attention		Fleischer et al. (30)		Limited evidence of reduced risk of HRI
**Sociodemographic factors**				
**Gender**				
Female gender	Mac (79) Mac et al. (74) Mutic et al. (57) Kiatkitroj et al. (69)		Riccò et al. (63) Vega-Arroyo et al. (75)	Suggested evidence of increased risk of HRI
Male gender	Grimbuhler and Viel (85)			
Older age	Arcury et al. (60) Arcury et al. (49) Arnold et al. (51)	Spector et al. (27)	Mutic et al. (57) Riccò et al. (63) Vega-Arroyo et al. (75) Mac et al. (74) Grimbuhler and Viel (85)	Inconsistent or conflicting evidence
Migrant status			Riccò et al. (63) Mutic et al. (57) Arcury et al. (49) Arnold et al. (51)	no evidence from increased risk of HRI
**Environmental conditions**				
Hot environmental conditions	Vega-Arroyo et al. (75) Gun and Budd (72)		Mac et al. (74)	Suggested evidence of increased risk of HRI
	Grimbuhler and Viel (85) Ioannou et al. (87) Mohammadian et al. (84)			

*Suggested evidence: Two or more of the reviewed studies identified the same risk or protective factor*.

*Limited evidence: Only one study identified the risk or protective factor*.

*Inconsistent or conflicting evidence: One or more studies identified a specific risk or protective factor while one or more studies identified a contradictory association or a non-significant association*.

*No evidence: No studies identified a significant association*.

* *criteria used to evaluate the risk factors*.

###### Job Decision Latitude.

Two qualitative studies identified that a lack of control over workplace conditions prevented agricultural workers from accessing HRI preventative measures, such as clean drinking water, shade, and bathroom facilities (29, 103).

###### Work Safety Climate.

One qualitative study reported through a case study that migrant forestry workers employed by companies with a poor work safety climate were less likely to obtain adequate treatment for various injuries including HRI and to achieve full recovery (104).

##### Behavioral Factors

In the reviewed studies, various behavioral factors were identified as protective against heat stress, including drinking water (69), reducing soda consumption (30), and changing work hours and activities (40) (Table 3). One study identified that wearing excessive clothing is associated with greater odds of reporting HRI (69). In contrast, other behavioral factors assessed in the reviewed studies such as sun safety behavior and HRI training were reported to have no significant association with HRI symptoms (27, 30, 40, 55, 63).

###### Accessibility to Heat Prevention and Treatment.

Various risk factors related to accessibility to heat prevention and treatment were identified in the reviewed studies (Table 3). One study identified that a lack of access to bathroom facilities was a risk factor for HRI (27), while another reported that greater access to medical attention was protective for HRI (30). Furthermore, two qualitative studies identified that cultural and language barriers could increase the risk of HRI by preventing workers from understanding proper safety training requirements (29) or interfering with HRI treatment (103).

##### Sociodemographic Factors

###### Age.

Three studies identified increasing age as a risk factor for HRI among child farmworkers (49, 51, 60). However, evidence regarding the effect of age on HRI among adult farmworkers was inconsistent between studies. One study reported that increasing age was associated with 0.92 times lower odds of HRI among crop workers (OR: 0.92; 95% CI: 0.87–0.98) (27). In contrast, some studies found that younger workers were more likely to have higher HRI, although the association between age and HRI was not significant (57, 63, 74, 75).

###### Gender.

Four studies identified female gender to be associated with increasing HRI (Table 3) (57, 69, 74, 79). Two studies found that female fernery workers were 5.38 times more likely to have core body temperature reaching or exceeding 38°C as compared to males after adjusting for energy expenditure (OR: 5.38, 95% CI: 1.03–18.30) (74, 79). In contrast, other studies found no significant association between female gender and HRI (63, 75). In addition, one study identified that male gender was associated with increased mean skin temperature among vineyard workers (β: 1.447; 95% CI: 0.922–1.972) (85).

###### Migrant Status.

Several studies found no association between migrant status or nationality and increased risk of HRI (49, 51, 57, 63).

###### Environmental Conditions.

Five studies identified a strong association between HRI and hot environmental conditions as measured by WBGT (Table 3) (72, 75, 84, 85, 87). Vega-Arroyo et al. (75) reported that WBGT was associated with higher core body temperatures (β: 0.03, 95% CI: 0.017–0.05) among migrant farmworkers. In addition, dry bulb temperature was associated with an increase in cardiac strain, including mean heart rate (β: 4.084; 95% CI: 0.442–7.726), relative cardiac cost (β: 3.652; 95% CI: 0.888–6.416), and cardiac workload score (β: 0.644; 95% CI: −0.046–1.334) among vineyard workers (85). However, Mac et al. (74) found that the average WBGT was not a significant predictor of elevated core body temperatures among fernery workers.

#### Factors Associated With Kidney Disease

##### Work Organization and Management

###### Workload.

Two studies conducted in the US reported a positive association between the workload, measured using an accelerometer, and cross-shift AKI among agricultural workers; the reported odds ratios ranged between 1.01 and 1.92 (Table 4) (93, 95). In addition, several studies showed that workers performing the most strenuous tasks had a greater decline in kidney function over a harvest season as compared to workers conducting less strenuous tasks (Table 4) (93, 96, 105, 106). Hansson et al. (100) found that burned cane cutters and seed cutters (who conduct more strenuous tasks) had significantly lower cross-harvest eGFR and higher IKI as compared to field support staff and drip irrigation workers. Similarly, Laws et al. (105) found a significant increase in kidney injury biomarkers (NGAL, NAG, and IL-18) among field workers (cane cutters, seeders, seed cutters, agrichemical applicators, and irrigators) as compared to non-field workers (drivers and factory workers).

**Table 4:** Risk and protective factors associated with kidney diseases.

**Factors**	**Risk factor**	**Protective Factor**	**No significant Association**	Comment^*^
**Work organization and management**				
Workload	Hansson et al. (100) Moyce et al. (95) Moyce et al. (93) Laws et al. (105) Laws et al. (106) Kupferman et al. (96) Moyce et al. (93)			Suggested evidence of increased risk of kidney disease
Piece-rate payment	Moyce et al. (28) Moyce et al. (95) Moyce et al. (94)			Suggested evidence of increased risk of kidney disease
**Physiological factors**				
Dehydration	Butler-Dawson et al. (92) Sorensen et al. (107) López-Gálvez et al. (108)		Moyce et al. (28)	Suggested evidence of increased risk of kidney disease
Heat strain	López-Gálvez et al. (108) Moyce et al. (28)			Suggested evidence of increased risk of kidney disease
**Behavioral factors**				
**Fluid consumption**				
Increased fluid consumption	Moyce et al. (93)	García-Trabanino et al. (109)	Wesseling et al. (110) López-Gálvez et al. (108)	Inconsistent or conflicting evidence
Lower fluid consumption		Pundee et al. (111)		
Electrolyte intake		Butler-Dawson et al. (92) Hansson et al. (99) Laws et al. (105) Laws et al. (106) Wesseling et al. (110)		Suggested evidence of increased risk of kidney disease
Sugary beverage intake	Hansson et al. (99) Raines et al. (112)		Butler-Dawson et al. (113) Butler-Dawson et al. (92) Moyce et al. (93)	Inconsistent or conflicting evidence
**Medications and lifestyle factors**				
NSAIDs use	Butler-Dawson et al. (92) Hansson et al. (99)		Sorensen et al. (107) Wesseling et al. (110)	Inconsistent or conflicting evidence
**Sociodemographic factors**				
Female gender	Moyce et al. (28) Jayasekara et al. (114)			Suggested evidence of increased risk of kidney disease
Socioeconomic status	Jayasekara et al. (114)			Limited evidence of increased risk of kidney disease
**Environmental conditions**				
Agrochemical exposure	Raines et al. (112) García-Trabanino et al. (109) López-Gálvez et al. (108)		Laws et al. (105) Ekiti et al. (91) Fitria et al. (90) Wesseling et al. (110)	Inconsistent or conflicting evidence
Hot environmental conditions	García-Trabanino et al. (109) Raines et al. (112) Sorensen et al. (107) Mix et al. (97)			Suggested evidence of increased risk of kidney disease

*Suggested evidence: Two or more of the reviewed studies identified the same risk or protective factor*.

*Limited evidence: Only one study identified the risk or protective factor*.

*Inconsistent or conflicting evidence: One or more studies identified a specific risk or protective factor while one or more studies identified a contradictory association or a non-significant association*.

* *criteria used to evaluate the risk factors*.

###### Payment Method.

Three studies conducted in the US identified piece-rate work as a risk factor for cross-shift AKI among agricultural workers with an odds ratio ranging from 3.02 to 4.52 (Table 4) (28, 94, 95).

##### Physiological Factors

###### Dehydration.

Evidence from 3 studies suggested that dehydration was a risk factor for the development of kidney disease among agricultural workers (Table 4) (92, 107, 108). One of these studies found a significant decrease in eGFR of 11.99 mL/min/1.73 m^2^ (95% CI: −16.88, −7.10) among dehydrated farmworkers when compared to those who were normally hydrated (108). However, one study did not find an association between dehydration and kidney disease (28).

###### Heat Strain.

Two studies found a significant association between heat strain that was measured using the physiological strain index and the decline in kidney function (28, 108). López-Gálvez et al. (108) found that for every 1% increase in PSI, there was a significant decrease in eGFR of 0.16 mL/min/1.73 m^2^ (95% CI: −0.15, −0.06).

##### Behavioral Factors

###### Fluid Consumption.

Four studies assessed the association between fluid consumption and kidney disease among agricultural workers (Table 4) (28, 107–109, 111). Overall, the studies showed conflicting evidence regarding the effects of fluid consumption on kidney disease. Two studies showed that an increase in fluid consumption was beneficial for preventing kidney disease (109, 111). In contrast, two studies found that fluid consumption was not associated with reduced kidney function (108, 110).

###### Electrolyte Consumption.

Five studies conducted in Central America identified an association between greater electrolyte intake and improved kidney function (Table 4) (99, 105, 106, 110, 113). Butler-Dawson et al. (92) showed that lower electrolyte intake was associated with higher odds of AKI among sugarcane workers in Guatemala (OR: 0.94, 95% CI: 0.89–0.99). Similarly, Hansson et al. (99) showed that electrolyte consumption was associated with lower IKI (IR: 0.5, 95% CI: 0.2–0.9). In addition, three studies showed that increasing electrolyte consumption was associated with improved kidney function biomarkers (105, 106, 110).

###### Sugar Consumption.

Two studies conducted in Central America reported that sugar consumption was a risk factor for kidney diseases (Table 4) (99, 112). Raines et al. (112) identified that daily bolis consumption, which is a sugary rehydration packet, was associated with reduced eGFR (OR: 1.48, 95% CI: 1.02–2.14) (112). In contrast, other studies found no association between intake of sugary beverages and kidney disease (Table 4) (92, 93, 113). Overall, evidence regarding the effect of sugar consumption on the decline in kidney function was inconsistent.

##### Medications and Lifestyle Factors

Two studies in Central America identified non-steroidal anti-inflammatory drugs (NSAIDs) use as a risk factor for kidney disease among sugarcane workers (Table 4) (92, 99). One of these studies found that dehydration in combination with NSAIDs use was associated with higher odds of AKI (OR: 8.38, 95% CI: 1.67–42.16) (92). However, three other studies found that NSAIDs use was not significantly associated with kidney dysfunction among sugarcane workers (100, 107, 110). Overall, the evidence regarding the effect of NSAIDs on the development of kidney disease remains inconsistent.

##### Sociodemographic Factors

The sociodemographic factors that were identified as risk factors include gender and poor socioeconomic conditions. Jayasekara et al. (114) found a significant correlation between higher income and lower heat stress-dehydration symptom scores among agricultural workers in India, indicating that poor socioeconomic conditions can increase the risk of kidney disease. In addition, evidence from the study suggested an increased risk of kidney disease among females (28, 114).

##### Environmental Conditions

###### Hot Environmental Conditions.

Five studies identified hot environmental conditions as a risk factor for reduced kidney function. Three of these studies were conducted in Central America among sugarcane workers (Table 4) (107, 109, 112). One of these studies identified that an increase in the average WBGT by 1°C was associated with a 1.6% decline in cross-shift eGFR. In addition, two other studies were conducted in the U.S. focusing on the association between hot environmental conditions and cross-shift AKI among agricultural workers (28, 97). Mix et al. (97) found that each 5°F increase in mean heat index was associated with a 47% increase in the likelihood of AKI among migrant agricultural workers.

###### Agrochemical Exposure.

Evidence regarding the effect of agrochemical exposure on the development of kidney disease remains conflicting. Two studies conducted in Central America found a significant association between agrochemical exposure and the decline in eGFR (Table 4) (40, 109). Moreover, one study conducted in Mexico reported that farmworkers, who are more likely to be exposed to pesticides, experienced a greater decline in eGFR (β: −29.17; 95% CI: −37.26, −21.17) (Table 4) (108). In contrast, other studies conducted in Central America (105, 110) and nonendemic regions (90, 91) did not find a significant association between pesticide use and kidney disease (Table 4).

#### Vulnerable Populations

##### Migrant Farmworkers

Twenty-two studies focused specifically on migrant farmworkers in the U.S. (30, 40, 49–52, 55–58, 60, 78, 81, 82, 101, 104, 108, 115–118), Mexico (108), Ethiopia (65), and Australia (29). Three of these studies were focused on migrant Latino farmworkers, who have changed residence from one geographic location to another in search of agricultural work (49, 51, 60). According to the results of the reviewed studies, migrant farmworkers experience high levels of HRI symptoms with HRI prevalence ranging between 18 and 79% (30, 52, 55, 56, 58, 115). In addition, one study found that 33% of migrant agricultural workers in the U.S. experienced AKI (97). Only a few studies assessed the risk and protective factors associated with heat-related health outcomes among migrant farmworkers (30, 40, 50, 55, 97, 108). Three studies identified several risk factors to be unique to migrant agricultural workers, including lack of control over workplace conditions, poor work safety climate, and cultural and language barriers (29, 103, 104). Another risk factor, common for both migrant and non-migrant agricultural workers, was hot environmental conditions (97).

##### Child Farmworkers

Child farmworkers were the target population in 4 studies that examined the experiences, risk factors, and preventive measures associated with HRI (49–51, 60). These studies reported that child farmworkers faced a poor work safety climate and often lacked access to healthcare, sanitation facilities, and proper heat prevention measures (49–51, 60). HRI prevalence was high in this population with nearly half of the child farmworkers reporting at least one HRI symptom in the previous 12 months according to one study (51).

#### Identified Gender Differences in Health Outcomes and Associated Factors

Two studies used gender stratification to compare risk factors for kidney disease between males and females (28, 93). One of these studies found an association between kidney disease and (1) piece-rate work (OR: 102.81, 95% CI: 7.32–1443.20) and (2) years working in agriculture (OR: 1.12, 95% CI: 1.01–1.24) among female agricultural workers (28). Meanwhile, heat strain was associated with higher odds of AKI among males (OR: 1.31, 95% CI: 1.01–1.70). The second study found that picking or harvesting crops was a risk factor for kidney disease among males (OR: 4.12, 95% CI: 1.87–9.08) (93).

One of the reviewed studies compared HRI symptoms between male and female agricultural workers and identified several symptoms to be more prevalent among females than males, including headache, exhaustion, fainting, fatigue, and nausea or vomiting (62). Another study found that the prevalence of HRI symptoms among male and female sugarcane farmers was 30.4 and 59.5%, respectively (69).

#### Preventive Measures

The most common preventive measures identified in the included studies were maintain proper hydration, taking breaks in shaded areas, going to air-conditioned places during or after work, changing work hours and activities, and taking extra breaks (40, 50–52, 62, 63, 116). Water was found to be the most commonly consumed beverage in addition to sports drinks, energy drinks, soda, fruit juice, and coffee to maintain hydration (56, 61, 97, 116). Some studies also reported that the consumption of electrolyte solutions among agricultural workers reduced heat stress (98, 101). Several studies also reported the practice of wearing head protection such as baseball caps, hats, bandannas, and hoods from sweatshirts as a means of reducing heat stress (52, 55, 56, 63). In addition to head protection, wearing different types of clothing were used to protect against heat stress. These included wearing long-sleeved shirts, long pants, and light-colored or lightweight shirts (52, 55, 56, 62, 101, 116). Other commonly used preventive measures include acclimatization (52, 116), HRI training (63, 116, 117), wearing sunglasses and sunscreen (55, 63), and the use of rest stations or fans (56, 116). Preventative measures that were less common among the reviewed studies included eating a traditional diet (119) and bathing in cold water (53, 116).

## Discussion

This is the first scoping review that aims to describe the health outcomes, risk factors, and preventative measures related to occupational heat stress among agricultural communities globally. The most common heat-related health effects identified in this review were HRI and kidney disease. The majority of the reviewed studies were conducted in Central and South America.

### Factors Associated With HRI

Various risk factors associated with HRI were identified in the reviewed studies, including gender, workload, piece-rate payment, job decision latitude, cultural and language barriers, and hot environmental conditions. Although the evidence was limited, other risk factors that were found to have some form of association with HRI were reducing soda consumption and increasing access to regular breaks.

In addition, various work organization and management factors were associated with increased risk of HRI among agricultural populations, including workload and piece-rate payment. Several studies have shown that agricultural workers frequently perform strenuous physical activities under that payment method (97, 120). While piece-rate payment may incentive agricultural workers to work faster, it may also cause the workers to take fewer breaks to rest or to drink water (103, 120–122). However, some argue that adopting a piece-rate payment system can allow farmworkers to modify their work effort more easily during different heat exposure levels as compared to workers that are paid an hourly wage (123).

Another risk factor related to work organization and management was the lack of job decision latitude in agricultural workplaces. This results in a lack of control over certain HRI preventative measures such as shade availability and proximity to bathroom and water facilities (30, 116). This is likely due to the presence of a power imbalance between employers and agricultural workers, which prevents workers from making decisions about their health and safety or complaining about poor working conditions. In addition, migrant farmworkers often strive to keep their jobs at any cost, particularly since their legal status is tied to their employment (36).

A limited number of articles from the reviewed studies showed that various behavioral factors can be protective against HRI, such as taking breaks in the shade and increasing access to regular breaks. According to the Occupational Safety and Health Administration (OSHA) guidelines, workers should rest for 15 min each hour when WBGT exceeds 26°C, 30 minutes of rest each hour when WBGT reaches 28°C, and 45 min of rest each hour when WBGT reaches 30°C (124). However, the loss of working hours can reduce productivity and affect income and food security in agricultural workers and their families (125). On the other hand, studies have shown that improved access to rest breaks combined with an efficient intervention program can be effective in reducing heat-related health outcomes with minimal impacts on food production (59, 126).

In addition, a few studies suggested that reducing soda consumption can be protective for HRI among farmworkers. In fact, soda consumption is common among agricultural workers, who drink it to increase alertness and productivity (56, 61, 97, 103, 116). According to OSHA guidelines, workers should be encouraged to choose water over soda and other drinks containing caffeine and high sugar content since these products can increase the risk of dehydration (127).

Furthermore, several risk factors related to accessibility to heat prevention measures and treatment have been identified. One of these risk factors is cultural and language barriers that can affect the prevention and treatment of HRI among farmworkers. These language and cultural barriers prevent migrant farmworkers from understanding health and safety information (128–130) or accessing health care services (129).

Findings from the reviewed studies indicated that female agricultural workers were more likely to experience HRI as compared to their male counterparts. This may be due to lower water consumption, as females lack access to sanitation facilities when working in agricultural settings (131) and are less comfortable taking breaks to drink water (117). However, the research shows that agricultural tasks are often segregated by gender with the more strenuous tasks being performed by men, consequently increasing their risk of HRI (121).

However, the effect of other demographic variables such as age and migrant status were either inconclusive or limited. The reviewed articles show conflicting evidence concerning the association between age and HRI among adult farmworkers. Older farmworkers are more likely to be knowledgeable about HRI preventative measures and treatment due to greater experience (27). However, other sociodemographic factors, such as migrant status, were reported to be associated with HRI but were not identified as risk factors in the reviewed studies (30, 50, 52).

Another risk factor for HRI that was identified in the reviewed studies was hot environmental conditions. Therefore, occupational health agencies recommend various preventative measures that can be implemented to reduce heat exposure while working in hot conditions. OSHA has established four risk categories based on heat index values (127). OSHA recommends that preventative measures should be a function of the risk category.

### Factors Associated With Kidney Disease

Various risk factors associated with HRI were identified in the reviewed studies, including gender, workload, piece-rate payment, and hot environmental conditions. Meanwhile, electrolyte consumption was identified as protective for kidney disease. Although the evidence was either limited or conflicting, other risk factors that were found to have some form of association with kidney disease included sugary beverage intake, fluid consumption, NSAIDs use, and poor socioeconomic conditions.

Various factors related to work organization and management have been identified as risk factors for kidney disease. Evidence from the studies identified in this review suggests that heavy workload is significantly associated with kidney diseases. In addition, the reviewed studies indicated that sugarcane cutters had a higher prevalence of reduced kidney function. Sugarcane cutters required unusually high physical exertion and fast repetitive movements with a machete (132, 133). In addition, the findings of this review suggest that piece-rate work is significantly associated with kidney diseases among agricultural workers. This payment method can cause agricultural workers to push themselves beyond their physical limits to earn as much as they can and to take fewer breaks to rest or drink water leading to a higher risk of heat stress and dehydration and consequently to the development of kidney disease.

Evidence from the reviewed studies indicates that several physiological factors including dehydration and heat strain are associated with kidney disease. Similarly, research shows that dehydration due to the loss of body water is well known to be associated with chronic kidney disease (134). In addition, heat strain resulting from heavy physical workload and hot environmental conditions can lead to kidney injury (99, 135).

An important behavioral factor that can affect the development of kidney diseases among agricultural workers was the adopted hydration practices. Evidence from the reviewed studies suggests that the intake of electrolyte solutions can be protective against kidney disease. According to the US National Institute for Occupational Safety and Health (NIOSH), electrolyte replacement should be provided to workers in the form of sports drinks containing balanced electrolytes if excessive sweating occurs for 4 or more hours in hot conditions (12). On the other hand, OSHA guidelines recommend water consumption but do not comment on electrolyte replacement (127). However, various studies reported that agricultural workers consumed low amounts of electrolyte solutions (92, 105).

Furthermore, our findings suggest that rehydration with sugary beverages may be a risk factor for kidney disease among agricultural workers, although the current studies remain inconclusive. The literature shows that sugary beverage intake has been reported as a risk factor for kidney disease in several experimental studies (136). Similarly, the effect of fluid consumption on the development of kidney disease has also been contradictory across the reviewed studies. In fact, the literature has reported that the fluid consumption of agricultural workers was often below the recommendations of occupational health agencies and consequently resulted in a greater risk of kidney disease (58, 69, 93, 111).

Evidence from the reviewed literature regarding the effect of NSAIDs use on kidney disease was found to be contradictory. However, the literature indicates that recurrent dehydration or volume depletion can be exacerbated by NSAIDs use (137, 138). In addition, a systematic review published in 2018 indicated that NSAIDs were found to be significantly associated with CKDu in Mesoamerica (139).

Various sociodemographic factors were also identified as risk factors for kidney disease in the current review. Findings from the reviewed studies suggest that female agricultural workers are at a greater risk of kidney disease as compared to their male counterparts. However, two systematic reviews identified in the literature showed a positive association between males and CKDu (139, 140). In addition, a limited number of studies indicated that low socioeconomic status is associated with an increased risk of kidney disease. This is consistent with the literature, which indicates that CKDu is mainly concentrated in poor agricultural communities characterized by heavy use of agrochemicals, hot and humid environments, and harsh working conditions at agricultural workplaces (141).

The majority of studies suggest that hot environmental conditions are significantly associated with kidney diseases, especially among sugarcane workers in Central America. This is consistent with the findings reported in the literature that indicate that occupational heat stress is a primary factor in the development of CKDu (43, 142, 143). In fact, various studies found that sugarcane workers in Central America are exposed to high levels of heat exposure (24, 109).

Another environmental factor related to kidney disease is agrochemical exposure. The majority of the reviewed studies suggest a lack of association between pesticides and kidney disease among agricultural populations. Similarly, a systematic review found no significant association between pesticides and CKDu in agricultural workers in Mesoamerica (139). Yet, another systematic review recommended that better exposure assessments are needed before ruling out pesticides as a cause of CKDu, since the past studies did not examine biomarkers of agrochemical exposure and instead relied only on self-reports of work practices and history (144).

### Vulnerable Populations

The reviewed literature indicates that migrant agricultural workers are at particularly high risk for heat-related health outcomes, including HRI and kidney disease. Our results also showed that some of the risk and protective factors can be unique and distinct to the migrant agricultural workforce. Moreover, migrant agricultural workers face several barriers that prevent them from adhering to appropriate heat prevention measures or seeking proper treatment including lack of control over workplace conditions, poor work safety climate, and cultural and language barriers (29, 103, 104). In addition, various studies have reported that migrant farmworkers lack access to common heat preventative measures including prevention training, regular breaks, areas with shade, and drinking water (30, 52, 55, 116). Furthermore, few studies have assessed heat-related health effects among child farmworkers. However, child farmworkers face various physiological and behavioral differences that raise their vulnerability to heat stress as compared to their adult counterparts (145). Further research is still needed to determine the prevalence of heat-related health effects as well as to determine the risk and protective factors among these vulnerable workers so as to design more effective preventative measures tailored to these populations. In addition, there is a need for paying a greater attention to the impacts of the poor working conditions and the high poverty levels that face these farmworkers so as to better understand their vulnerabilities to heat-related health effects.

### Gender Differences in Health Outcomes and Associated Factors

Piece-rate payment and years working in agriculture were identified as risk factors for kidney disease among females (28). Female agricultural workers, who are paid by piece, have an additional incentive to avoid visiting the bathroom during their work shift. Other reasons include the lack of adequate sanitation facilities in agricultural workplaces and the risk of sexual harassment or assault, which usually occurs around bathroom facilities. As a result, women limit their water consumption which leads to an increased risk of kidney disease (28). In addition, women working for longer durations in the agricultural sector experience frequent chronic delayed urination, which increases their risks of developing kidney diseases (28). Moreover, males are more likely to perform strenuous tasks such as picking or harvesting, which increases their risk of heat stress (28, 95).

However, the reviewed studies have not thoroughly investigated the differences in the prevalence of heat-related health effects and associated risk factors between male and female farmworkers. Since it is well established that men and women perform distinct agricultural tasks and have different occupational exposures even when given a similar job classification in studies (37, 117, 146), the use of a gender-sensitive approach in research is necessary to identify specific gender risk factors (147). Future studies should also compare the prevalence of heat-related health outcomes among male and female agricultural workers to gain a greater understanding of gender differences in vulnerability to heat stress.

### Preventive Measures

Various heat preventive measures practiced by agricultural workers have been identified in the reviewed studies. The most common administrative preventive measures included drinking more water, taking breaks in shaded areas, going to air-conditioned places during or after work, changing work hours and activities, and taking extra breaks. In addition, a few studies showed that agricultural workers practice acclimatization by gradually increasing the number of work hours at the start of the season, which is an important protective measure for HRI. OSHA has recommended the “Water. Rest. Shade.” guidelines to reduce heat stress in hot working environments (148). Furthermore, a systematic review has also identified similar preventative measures practiced by outdoor workers (149).

In addition, several studies identified various sun safety behaviors commonly used by agricultural workers, including clothing practices such as wearing long-sleeved and light-colored shirts and long pants. Furthermore, several studies reported that agricultural workers also use sunscreen and sunglasses as well as head protection such as baseball caps, hats, bandannas, and hoods. NIOSH recommends that workers should wear breathable, light-colored, and loose-fitting clothing when working in hot environmental conditions (150), while OSHA encourages workers to use sunscreen and other protections from direct sunlight such as wearing hats (127).

On the other hand, several reviewed studies showed that agricultural workers lack access to adequate cooling measures such as shade, water, sanitation facilities, HRI training, and rest breaks (30, 55, 116). Furthermore, workplace culture can prevent the effective implementation of these preventive measures (29, 103).

### Research Limitations, Literature Gaps, and Implications for Future Research

The findings of this review demonstrate that there is limited information on health outcomes related to occupational heat stress among agricultural workers globally. The US represents the country with the highest number of HRI studies. In addition, the majority of studies assessing the prevalence of kidney disease have been concentrated in Central America. However, various hot regions such as Sub-Saharan Africa, the Middle East and North Africa, and Southeast Asia host millions of vulnerable agricultural workers and thus the need for studies assessing the impacts of hot temperatures on health outcomes in these populations is imperative (151, 152). Therefore, more studies are required globally to quantify the burden of heat-related health effects, especially as working conditions and environmental heat exposure often vary from one country to another.

In addition, most studies examining the role of heat stress and dehydration on the development of kidney disease have been concentrated in Central America. Since the adverse impacts of heat stress on kidney function is expected to increase due to rising global temperatures, future studies should focus on other agricultural communities exposed to hot environmental conditions to gain a better understanding of the relationship between occupational heat stress and kidney health and its interaction with other possible etiologic factors. Furthermore, evidence regarding the effect of high temperatures on various health outcomes, such as occupational injuries and mental health, was found to be limited in the reviewed studies (153–155).

Limited evidence was found in the current review on the potential effects of various risk or protective factors such as acclimatization status, hydration practices, training, migrant status, and poor socioeconomic conditions on HRI and kidney disease prevalence. Therefore, future studies should investigate in more detail these factors. In addition, there is a need for qualitative studies that can provide a critical social lens for the analysis of heat-related health outcomes in the vulnerable farmworker populations. These can improve our understanding of the role that these factors have regarding HRI so that effective policies and programs can be developed. Another important limitation identified in the reviewed studies was the reliance on cross-sectional design to assess the association between potential risk factors and HRI. Various behavioral factors, which are protective for HRI, have been identified as risk factors. Therefore, longitudinal studies would be better equipped to assess workers' risk of occupational heat stress and can also address reverse causality.

### Improved Methods of Exposure Assessment

Greater consistency in assessment tools used to examine heat-related health outcomes among farmers may be beneficial for future research. The identification of risk or protective factors associated with heat stress or kidney disease requires the design of consistent and comparative multisite studies in high-risk populations. Most studies assessing the prevalence of HRI relied mainly on self-reported HRI symptoms, using questionnaires that have not been validated. Comparison of HRI symptoms between studies is difficult due to different reporting periods, number of symptoms, and case definitions of HRI. Instead, heat exposure could be better assessed using validated questionnaires that can be modified and applied to local conditions, such as the HOTHAPS (The High Occupational Temperature, Health, and Productivity Suppression) questionnaire was used in some of the included studies (25, 62). In addition, objective measures of HRI are needed to provide more valid estimates of the prevalence of heat stress and dehydration among agricultural populations. These include measuring core body temperature through the use of an ingestible temperature pill (75, 78, 79, 95). Future research should also define objective measures of physical activity using accelerometers (156) or heart rate monitors (157) to measure the heat load generated by the performance of specific agricultural jobs.

### Policies, Regulations, and Recommendations

In light of the existential and global threat of climate change, outdoor workers including agricultural workers are at the forefront of exposure to occupational heat stress (149, 158). It is critical that policymakers design labor protection and occupational health and safety policies to protect vulnerable agricultural communities from the effects of climate change, including heat stress (159). Furthermore, current and future heat protection standards and guidelines have to be developed in a manner that ensures that they are suitable for local environmental conditions and the physical requirements of the agricultural tasks involved. Thus, farm managers or supervisors ought to implement appropriate administrative and engineering measures such as the provision of water, shade, and rest breaks as well as provide better education and training instead of relying on the voluntary will of workers to implement heat preventative measures. Moreover, employers ought to develop and implement health surveillance programs that can detect early physiological responses of HRI among vulnerable workers (127, 160). In addition, implementing health surveillance can also provide evidence about the effectiveness of different preventive measures (160). Furthermore, it is necessary to work with various stakeholders throughout the global food supply chain to strengthen labor protections and ensure compliance with regulations. High-income countries could join effort with low- and middle-income countries to implement adaptation measures in the agricultural sector that protect the health and safety of farmworkers and ensure their food security (10, 150). Greater mechanization can be introduced in labor-intensive activities to reduce the level of physical activity required by workers thereby reducing heat stress (87). Furthermore, farming activities can be modified to reduce the risk of excessive exposure to heat by changing the timing of work activities or implementing best practices to reduce the time and work intensity needed (161). Other measures include monitoring of local weather conditions to plan agricultural activities accordingly (19).

However, commonly recommended administrative measures are sometimes ineffective in reducing the risk of heat stress among agricultural workers, especially as global temperatures continue to rise. Hence, various technological and engineering solutions should be developed to protect vulnerable agricultural communities from the effects of heat stress. Recent technological advancements have led to the rapid growth in the development of personal or microclimate cooling systems, such as personal cooling garments and cooling vests that can provide a convenient and flexible method to reduce heat stress and improve thermal comfort among working populations, including agricultural workers (87, 162–164). Other measures include various engineering controls including the use of mechanical aids to reduce the levels of physical activity (87, 165). However, these technical solutions may not be applicable in developing countries and marginalized communities due to their costs but could be easily implemented in developed countries. In addition, there is a need for field-based research to examine the effectiveness and practicality of these measures across different work settings and regions.

### Strengths and Limitations of the Current Review

To the authors' current knowledge, this is the first scoping review that aims to describe the prevalence, risk factors, and preventative measures of occupational heat stress among agricultural populations. All health outcomes related to occupational heat stress among agricultural workers were assessed. A wide range of literature formed the basis for this review as both peer-reviewed studies and gray literature published in various languages were included with no restriction on publication date. This scoping review can provide an overview of current research in the field and can be used as a starting point to guide future research to assess heat-related health outcomes among farmworkers.

However, similar to other scoping reviews, a limitation to this current scoping review is the lack of quality assessment of the included studies. Furthermore, the comparability of risk factors and prevalence of heat-related health effects between various regions and agricultural populations were difficult to perform due to the use of different measurement instruments and different groups of farmworkers in most studies.

## Conclusion

Given the projected increase in global temperatures and extreme heat events due to climate change, the adverse health impacts of occupational heat stress on agricultural communities are likely to increase in the future. This scoping review provides a comprehensive overview of heat-related health effects among agricultural populations as well as risk factors and preventative measures used to minimize heat stress exposure among farmworkers. Knowledge of risk factors and preventative measures is essential for reducing the burden of heat stress among agricultural workers. Thus, this scoping review is an important step toward synthesizing the knowledge base and highlighting areas for future research. The risk factors identified for both kidney disease and HRI include female gender, dehydration, heat strain, wearing inappropriate clothing, workload, piece-rate payment, job decision latitude, and hot environmental conditions. On the other hand, various protective factors were identified including drinking water, changing work hours and schedule of activities, reducing soda consumption, and increasing electrolyte consumption in addition to improving access to medical care. However, limited evidence was found regarding the effect of various factors such as acclimatization status, hydration practices, training, migrant status, and poor socioeconomic conditions on the prevalence of both HRI and kidney disease. The current review also identified various factors that are unique to vulnerable populations working in the agricultural sector, including migrant and child farmworkers. In addition, this review found that the current knowledge is largely based on findings from countries in North and Central America. Large gaps remain with regards to quantifying the burden of heat-related health outcomes among agricultural workers in hot regions such as Sub-Saharan Africa, the Middle East and North Africa, and Southeast Asia. These areas host millions of heat stress vulnerable agricultural workers that are understudied. Therefore, more studies are required globally to quantify the burden of heat stress and to identify understudied risk and protective factors among vulnerable agricultural communities in different geographical regions.

## Data Availability Statement

The original contributions presented in the study are included in the article/Supplementary Material, further inquiries can be directed to the corresponding author/s.

## Author Contributions

RH and ME: conceptualization. LH: database search strategy. ME, DH, and RH: literature review, screening articles and drafting early drafts and the manuscript. ME, RH, DH, IA, and MH: revising the final draft of the manuscript. All authors approved the final version of the manuscript.

## Conflict of Interest

The authors declare that the research was conducted in the absence of any commercial or financial relationships that could be construed as a potential conflict of interest.

## Publisher's Note

All claims expressed in this article are solely those of the authors and do not necessarily represent those of their affiliated organizations, or those of the publisher, the editors and the reviewers. Any product that may be evaluated in this article, or claim that may be made by its manufacturer, is not guaranteed or endorsed by the publisher.
